# Innovative and practical conditioning beverages for public health and athletic performance: Focus on immunopotentiation by lactic acid bacteria B240

**DOI:** 10.20463/jenb.2019.0011

**Published:** 2019-06-30

**Authors:** Minchul Lee, Kyunghee Kim

**Affiliations:** 1 Department of Sports Medicine, CHA University, Pocheon Republic of Korea; 2 Sports Science team, Marketing Department, Dong-a Otsuka, Seoul Republic of Korea

**Keywords:** conditioning beverages, immunity, B240, sports drink, ion water, BODYMAINTÉ

## Abstract

**[Purpose]:**

Functional beverages are a protective or enhancing factor influencing not only public health but also athletic performance. The purpose of this study was to highlight the new conditioning beverage of the Lactobacillus pentosus strain b240 (B240) with electrolytes or proteins, which strengthens immune functions to improve the quality of life.

**[Methods]:**

ISeveral related studies systematically reviews three main issues associated with conditioning beverages: (a) utilization and availability of the functional beverage; (b) significance of B240 in immune strengthening; and (c) availability and application of conditioning drinks in the daily life and sports field.

**[Results]:**

Intake of B240 led to greater enhancements, including blood T-helper, NK cell, IgA and IgG level in conjunction with strengthen immune func¬tions. These results speculated that the practical application of B240 contained beverages on physiological health and performance.

**[Conclusion]:**

BODYMAINTÉ, this novel conditioning beverage is expected biological utility responsible for improved sports performance as a functional drink and has potential health-related implications.

## INTRODUCTION

Functional beverages are drinks and food typically intended for health benefits, usually as a medicine or performance enhancer. Examples of functional beverages include sports drinks, energy drinks, and electrolyte (ion) water, with ingredients such as amino acids, vitamins, minerals, and additional fruits or vegetables^1^. These functional beverages have become popular among people who want specific health benefits and improved exercise performance. 

Functional beverages improve public health and athletic performance. They contribute to numerous benefits, such as hydration, maintenance of body fluid level, and improved physical condition. Moreover, these functional beverages help maintain optimum physical condition and raise the quality of life. For example, some functional beverages can improve heart health, immunity, and digestion, while others can supplement and boost energy. Utility and functionality have been identified as important factors influencing the choice of beverage.

Herein, we assess the efficacy of novel conditioning beverages containing the lactic acid bacteria B240 for performance improvement and physiological utility. In addition, we address the issues associated with the effects of B240 on improved immunity-related public health and athletic performance. 

### Novel conditioning beverages containing the lactic acid bacteria B240 to strengthen immunity

Conditioning drinks support the health and wellbeing of people who seek to maintain optimum physical condition in the daily life. Recently, Otsuka nutraceuticals formulated the proprietary *Lactobacillus pentosus* strain b240 (B240), which was named “BODYMAINTÉ.” This novel conditioning drink is a functional beverage that could provide protection from regular risks and support the health and wellbeing of people who seek to maintain an optimum physical condition.

B240 is a plant-derived lactic acid bacterium, which was discovered in the fermented tea "Miyan," traditionally consumed in northern Thailand. Otsuka nutraceuticals have long been contributing to local people's health through meat and bite teas, including the "Miyan" tea. It plays a role in maintaining optimum physical condition and prepares for risks on a daily basis^2^. 

Recently, B240 was shown to reinforce immune function, and the intake of Lactobacillus resulted in an increase in blood T-helper and natural killer (NK) cell counts and enhancement of NK cell activity in the elderly^3^. B240 is an anaerobic, non-speculating, gram-positive bacterium, originally isolated from fermented tea leaves^4^. Oral administration of B240 in mice resulted in increased synthesis of IgA in the mucosal tissue and increased serum IgG level^5,6^. Intake of B240 increases the secretion of salivary IgA in healthy adult women and in the healthy elderly^7,8^. Moreover, recent studies have shown B240-mediated IgA enhancement^9^. B240 is combined with other ingredients in novel products to support good physical condition based on the new concept of protective beverages.

### Two types of beverages: electrolytes and proteins 

In addition to consuming B240 in a basic and easy way, novel conditioning beverages are of two types, those containing electrolytes and those containing proteins. The former is in drinking form and offers an effortless way to consume the protective B240 and balance electrolyte concentrations for optimum physical condition^10^. It is similar to the body fluid to maintain hydration and is low in calories and refreshing^11^.

 The other is in jelly form containing B240 with the whey protein, citric acid and amino acids such as BCAA and Arginine, to help maintain physical condition against regular risks. This protein absorbs quickly and tends to incorporate in body tissues, such as muscles, which support the framework of the body^12^. Furthermore, essential amino acids play a role in muscle growth and recovery^13^. Moreover, arginine supports body recovery with various functions^14^. The purpose of these assorted drinks is to hydrate the body and improve athletic performance^15^. The jelly type is widely used by athletes and other people to maintain optimum physical condition and resilience.

### Appling conditioning beverages in the daily life and sports 

It is necessary to prepare for risks on a daily basis by having a good physical condition. People face many daily risks that could affect physical resilience, such as poor dietary habits, stress, and aging. People who heavily use the body, such as through sports, should firmly ensure maintenance of the body. Thus, maintenance of physical resilience is important for those concerned with living a healthy life. 

People regularly participate in strenuous daily activities, such as sports competitions or work presentations. Everyone wants to make the day a success through hard training and tough work. Therefore, physical condition management for productivity is important. About 60% of body fluid contains electrolytes, such as sodium, potassium, and calcium^16^. Therefore, beverages contain water and electrolytes close to the composition of the body fluid to maintain a balance between them in the body.

Athletes and people who exercise actively lose body fluid and electrolytes through sweat and expend energy. Exercising or competing in a sporting event for over one hour eliminates the need to drink something with excess sugar or electrolyte. Excessive salt supplementation during exercise could lead to gastrointestinal problems or cause further impairment of fluid balance and salt-induced cramps^17^. 

The general population occasionally has to work hard, which tends to put a burden on the body. Excessive stress and sleep deprivation make it more difficult to manage the physical condition. For such a person, B240 is recommended with electrolytes or proteins for immunopotentiation. It protects the body and maintains the daily physical condition for good productivity.

**Table 1.** Nutritional facts of the conditioning drink “BODYMAINTÉ” (drink type).

Nutrition Facts			
Per 100 ml			
Energy	18 kcal	Electrolytes	(mEq/L)
Protein	0 g	Na+	21
Fat	0 g	K+	5
Carbohydrates	4.4 g	Ca2+	1
Salt eqivarent	0.13 g	Mg2+	0.5
Potassium	20 mg	Cl-	16.5
Calcium	2 mg	citrate3-	10
Magnesium	0.6 mg	lactate-	1

**Table 2.** Nutritional facts of the conditioning food “BODYMAINTÉ” (jelly type).

Nutrition Facts			
Per pouch (100 g)			
Energy	90 kcal	Amino acid	2,500 mg
Protein	10 g	Valine	500
Fat	0 g	Leucine	1,000
Carbohydrates	13 g	Isoleucine	500
Salt eqivarent	0.11 g	Arginine	500
Vitamin B6	5 mg	Citric acid	1, 250 mg
Vitamin D	10 μg		

## CONCLUTION

In this article, we discussed the effects of the lactic acid bacteria B240 on body physiology. This novel conditioning beverage elicits biological utility responsible for improved sports performance as a functional drink and has potential health-related implications. Furthermore, we speculated that the practical application of B240 on performance is important in public health promotion, as lack of time is a common barrier to exercising regularly. However, the underlying mechanisms of B240 with electrolytes or proteins might differ from those seen in the subjects. Future studies are needed to uncover the usefulness and interventions for conditioning drinks in public health and athletic performance.
